# A Spectral Reflectance Model of Smooth Dry Soil Surfaces for Varied Soil Properties Based on Intelligent Learning

**DOI:** 10.3390/s26092765

**Published:** 2026-04-29

**Authors:** Jingwen Ma, Xiangdong Li, Xinxin Qiu, Zhuo Wu, Bingze Li, Xinbiao Li, Lulu Yan, Ranzhe Jiang, Si Chen, Nan Lin, Chunmei Wang, Zui Tao, Jianhua Ren, Yun Shi, Huibin Li, Xingming Zheng

**Affiliations:** 1 School of Geomatics and Prospecting Engineering, Jilin Jianzhu University, Changchun 130118, China; majingwen@iga.ac.cn (J.M.); chensi@jlju.edu.cn (S.C.); 2 Northeast Institute of Geography and Agroecology, Chinese Academy of Sciences, Changchun 130102, China; wuzhuo@iga.ac.cn (Z.W.); lixinbiao@iga.ac.cn (X.L.); 3 College of Information Technology, Jilin Agricultural University, Changchun 130118, China; lixiangdong@iga.ac.cn; 4 School of Remote Sensing and Information Engineering, North China Institute of Aerospace Engineering, Langfang 065000, China; qxx0323@stumail.nciae.edu.cn; 5 School of Biological and Agricultural Engineering, Jilin University, Changchun 130022, China; libingze@iga.ac.cn (B.L.); jiangrz23@mails.jlu.edu.cn (R.J.); 6 School of Geographical Sciences, Changchun Normal University, Changchun 130032, China; yanlulu@iga.ac.cn; 7 School of Modern Industry College, Jilin Jianzhu University, Changchun 130118, China; linnan@jlju.edu.cn; 8 Aerospace Information Research Institute, Chinese Academy of Sciences, Beijing 100194, China; wangcm@aircas.ac.cn (C.W.); taozui@aircas.ac.cn (Z.T.); 9 College of Geographical Sciences, Harbin Normal University, Harbin 150025, China; renjianhua@hrbnu.edu.cn; 10 Institute of Agricultural Resources and Regional Planning, Chinese Academy of Agricultural Sciences, Beijing 100081, China; shiyun@caas.cn (Y.S.); lihuibin@caas.cn (H.L.)

**Keywords:** dry soil, soil spectral, machine learning, SHAP

## Abstract

**Highlights:**

**What are the main findings?**

- A dry soil reflectance model (EEDSR) was developed based on an intelligent learning approach, with improved accuracy by incorporating parent material and geographic location.
- The applicability and generalization of EEDSR were validated globally using the ISRIC database.

**What are the implications of the main findings?**

- EEDSR supports accurate modeling of soil reflectance, effectively addressing simplifications in canopy radiative transfer models.
- The model provides technical support for precision agriculture and soil property inversion, enhancing the understanding of complex relationships between soil physicochemical properties and spectral characteristics.

**Abstract:**

Dry soil spectral reflectance provides a stable baseline for characterizing soil optical properties and supporting the retrieval of soil attributes from remote sensing. However, despite the large number of studies on soil spectral reflectance, most existing research primarily focuses on empirical relationships between spectra and soil properties. The representation and prediction of dry soil reflectance as a baseline condition, particularly under the influence of environmental factors, remain insufficiently explored, and the generalizability of existing models still needs improvement. Therefore, this study collects 700 dry soil samples with laboratory-measured spectral reflectance from Northeast China and quantitatively analyzes the contribution of environmental covariates (soil properties, parent material, and geographical location) using the SHAP method. Then, an environmental and edaphic-factor-driven smooth dry soil reflectance model (EEDSR) model covering 400–2500 nm is developed based on gradient boosting regression (GBR), and its accuracy is evaluated using global ISRIC soil datasets. Our results indicate the following: (1) the reflectance of dry soil is closely related to the soil properties in the VIS to SWIR range. The reflectance of dry soil of 400–2500 nm is positively correlated with clay percentage, longitude, and parent material but negatively correlated with latitude, sand percentage and silt percentage. And its correlation with other variables (such as soil organic matter, pH, and EC) varies with wavelength. (2) The EEDSR model exhibited high predictive accuracy across the 400–2500 nm spectral range (R^2^ = 0.93, RMSE = 0.018). Additionally, incorporating parent material (PM) and geographical factors into the predictor set enhanced the accuracy of dry soil reflectance prediction by 13.4%. (3) The spatial consistency between the predicted soil reflectance in Northeast China and the satellite observations indicates that the EEDSR model has good performance in predicting soil reflectance, as the bias of reflectance gradually increasing from west to east is consistent with the precipitation distribution in Northeast China. (4) The generalization ability of the EEDSR model was confirmed by global ISRIC datasets (R = 0.94), outperforming the deep learning-based Soil Optical Generative Model (SOGM) (R = 0.27). Overall, this study presents an efficient and interpretable framework for modeling dry soil spectral reflectance, providing a robust reference for soil reflectance prediction and remote sensing-based soil property retrieval.

## 1. Introduction

Soil spectral reflectance characterizes the ability of the soil surface to reflect incident radiation [1] and serves as a key parameter for revealing soil composition and surface properties [2]. The reflectance of soil provides valuable information on the soil’s mineralogical composition, textural structure, organic matter content, and salinity conditions, thereby reflecting its physical and chemical properties [3]. Accurate soil reflectance models support a wide range of applications, including the retrieval of soil physicochemical properties, assessments of land degradation, and monitoring of soil salinization [4,5]. Therefore, developing high-precision soil reflectance modeling approaches is of substantial importance.

Soil reflectance is jointly controlled by multiple factors, which can be broadly categorized into environmental factors, mineral composition, soil physical properties, and soil chemical properties [6].

Among the environmental factors, parent material (PM) reflects the pedogenic processes and material sources of a region and is recognized as an important soil-forming factor [7]. Parent material has been shown to affect soil composition and optical properties, which contribute to variations in shortwave infrared (SWIR) spectral behavior in arid and semi-arid environments [8]. Although parent material is not the sole or dominant control in soil formation, it remains an important explanatory factor for soil variability.

Observation conditions include observation geometry (incidence angle, viewing angle, and relative azimuth), terrain, weather and illumination [9]. Although these factors do not alter the intrinsic soil properties, they modify the interaction between light and the soil surface, affecting the shape of the spectral curve. As such, they represent important external contributors to spectral inconsistencies of the same soil under different observation scenarios [10]. Mineral composition is the primary determinant of absorption features in the near-infrared (NIR) and shortwave infrared (SWIR) regions. Clay minerals, carbonates, and iron oxides exhibit characteristic absorption positions, and their abundance and types—shaped by pedogenic environments—directly determine the depth and morphology of absorption features in the corresponding bands [11]. Among soil physical properties, surface roughness influences reflectance intensity and spectral behavior by altering light-scattering paths and reflection directions [12]. Soil moisture enhances spectral absorption and reduces scattering, leading to a general decrease in soil reflectance, with especially pronounced effects in the NIR-SWIR regions [13]. The proportions of clay, silt, and sand affect spectral responses mainly by modifying soil structure and porosity, thereby influencing reflectance in the visible (VIS) and red-edge bands through indirect mechanisms [14]. Regarding soil chemical properties, soil organic matter (SOM) exhibits strong absorption in the VIS region, substantially reducing reflectance [15]. Soil pH reflects chemical reactivity and affects the absorption characteristics of specific mineral functional groups [16]. Electrical conductivity (EC) can significantly modify reflectance in both the VIS and SWIR regions [17].

Methods for modeling soil reflectance can generally be categorized into three groups: empirical models, physical models, and intelligent learning models. Empirical models primarily describe the relationship between soil spectral reflectance and soil composition or observational conditions through statistical relationships or empirical functions. Among them, the Price model establishes empirical regression relationships between reflectance and soil compositional variables to characterize spectral variation patterns [18]; the General Spectral Vectors (GSV) model maps spectral reflectance into a vector space to represent spectral differences under different soil conditions [19]; and the Walthall model fits empirical functions to describe the relationship between reflectance, viewing geometry, and soil surface conditions [20].

Physical models are based on radiative transfer theory and the interaction between light and matter and simulate the formation process of soil reflectance from physical mechanisms. Specifically, the Hapke bidirectional reflectance model describes light absorption and multiple scattering processes at the particle scale to simulate soil reflectance characteristics [21,22,23,24]; the Multilayer Analytical Radiative Model of Inhomogeneous Targets (MARMIT) represents reflectance formation using a multilayer radiative transfer framework that accounts for contributions from different soil layers [25]; its improved version, MARMIT-2, further incorporates interface effects and more complex multiple scattering processes [26]; and the Bi-linear Soil Model (BSM) characterizes the combined influence of soil components on reflectance through a mixture of linear and nonlinear mechanisms [27].

Intelligent learning models establish nonlinear relationships between soil properties and spectral reflectance in a data-driven manner. The Soil Optical Generative Model (SOGM) uses machine learning methods to learn the complex mapping between input soil features and output spectral reflectance, enabling reflectance generation and prediction [28].

With the advancement of modeling methods, accurate simulation of dry soil reflectance is essential for improving the understanding of soil spectral mechanisms [29].

Compared with moist soil, the reflectance of dry soil more clearly reveals its intrinsic optical properties. Dry soil reflectance is widely regarded as a baseline parameter in soil spectral modeling and remote sensing applications [30]. It not only reflects the inherent information of soil composition and structure but also serves as a fundamental basis for simulating the spectral dynamics under varying moist conditions [31,32]. The empirical GSV model extracts three representative dry soil vectors through matrix decomposition to simulate dry soil spectra [19]. Based on empirical approaches, physical models further derive soil reflectance from radiative transfer theory by explicitly describing the interactions between light and soil particles. The Hapke model and its series of improved versions [21,22,23,24] are representative semi-empirical physical models that balance physical interpretability and computational efficiency. By incorporating optical constants (n and k), particle-size distributions, and geometric parameters, these models simulate multiple scattering and absorption processes to reconstruct the bidirectional reflectance of dry soils. Based on the Hapke framework, the SOILSPECT model was developed by introducing Legendre polynomials to approximate soil surface scattering characteristics [33]. Subsequent investigations using SOILSPECT examined the bidirectional reflectance behavior of soils under varying levels of surface roughness in both laboratory and field conditions [34]. With the advancement of artificial intelligence and the increasing availability of large-scale datasets, intelligent learning approaches—particularly machine learning and generative models—have been increasingly applied to soil reflectance modeling. Unlike traditional physical models, these methods do not rely on predefined physical equations; instead, they learn nonlinear mapping relationships directly from large collections of spectral samples. The BSM decomposes soil spectra into two key components—brightness and spectral shape—and reconstructs reflectance curves using empirically derived principal component functions obtained from extensive dry soil libraries, enabling efficient simulation of natural dry soil spectral variability with only a few parameters [27]. The SOGM further enables the generation of dry soil spectra conditioned on selected soil properties [28]. Despite the development of a range of dry soil reflectance simulation approaches, there remains a persistent challenge: the lack of direct and consistent mechanisms linking soil properties to reflectance, which limits the ability to achieve accurate pixel-scale simulations and broader remote sensing applications.

Most canopy radiative transfer models (such as PROSPECT [35], 4SAIL [36], and LESS [37]) typically simplify soil reflectance by using a specified soil spectral curve measured in the laboratory or obtained from spectral libraries. Such simplifications overlook the complex couplings between soil physicochemical properties (e.g., soil texture, SOM, salinity, and PM) and spectral reflectance. Consequently, they fail to capture the spectral variability of dry soils across different regions and soil types. Therefore, accurately characterizing the spatial variability of dry soil reflectance, and developing modeling frameworks that are both interpretable and capable of generalization, has become a major challenge in soil spectral modeling.

This study focuses on cropland soils in Northeast China and proposes an environment-driven intelligent prediction model (EEDSR). The model incorporates multiple sources of information, including soil physicochemical properties, spatial coordinates, and parent material, to characterize the spatial variability of dry soil reflectance. The predicted dry soil reflectance is intended to serve as a more informative input for canopy radiative transfer models—not only replacing conventional simplified spectral curves but also revealing the key soil properties that drive spectral variations, thereby achieving a unified understanding of spectral simulation and soil property characterization. Building on this, the dry soil reflectance serves as a benchmark for further simulating the spectral characteristics of wet soil under varying moisture conditions, supporting a comprehensive representation of soil spectral dynamics. Rather than treating these variables as independent features in a purely data-driven manner, they are interpreted as different levels of environmental controls on soil formation and spatial heterogeneity. Specifically, soil physicochemical properties represent local soil conditions, geographic coordinates capture spatial gradients, and parent material reflects pedogenic background information.

By integrating these multi-source environmental controls, the EEDSR model improves the accuracy and generalizability of soil reflectance simulation. It enables more physically informed modeling of soil spectral variability, alleviating the simplifications commonly adopted in current canopy radiative transfer models, and provides a reliable basis for simulating soil spectral responses under varying moisture conditions.

## 2. Data and Methods

### 2.1. Study Area

Northeast China was selected as the study area (117°46′–130°67′ E and 40°94′–49°25′ N). The region is characterized by a temperate monsoon continental climate, with a mean annual temperature ranging from 0 to 10 °C. Precipitation exhibits strong seasonality, with annual totals of approximately 400–1000 mm, of which 60–80% occurs during the summer months [38]. The major soil types in this region include chernozem, meadow soil, albic soil, and dark brown soil. The study area exhibits substantial pedological diversity, encompassing a wide range of soil types such as meadow soil, albic soil, calcic chernozem, chernozem, saline–alkali soil, and dark brown soil. The PMs are equally diverse and include dark and light crystalline rock weathering products, sandstone–shale complexes, limestone-derived weathering material, loose alluvium, neutral alluvium, lacustrine deposits, calcareous alluvium, coral reef detritus, and clay-rich loess. The pronounced spatial heterogeneity in soil types and PM provides a robust and representative foundation for this study.

### 2.2. Data

#### 2.2.1. Field Soil Data

(1) Collecting and processing of soil samples

**Figure 1:**
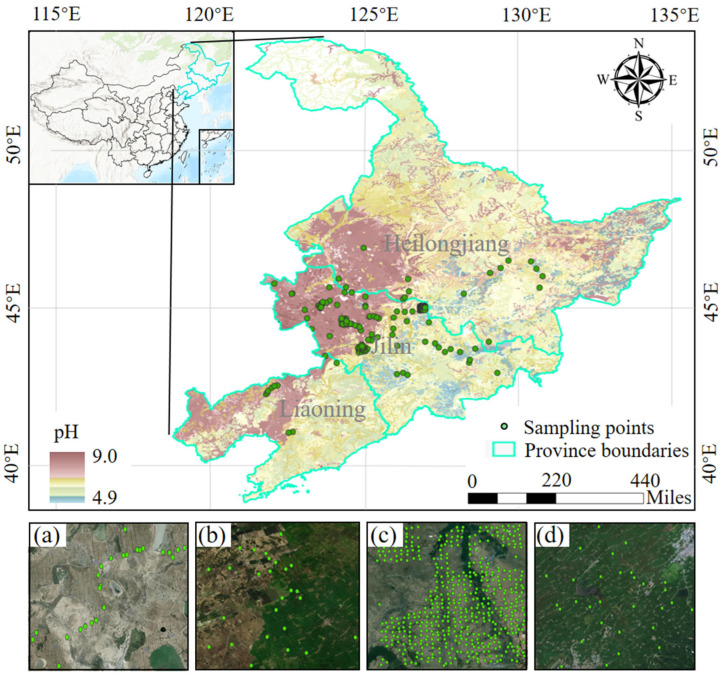
Spatial distribution of the study area and soil sampling points. The main map shows soil pH in Heilongjiang, Jilin, and Liaoning Provinces in Northeast China (pH 4.9–9.0). Green dots indicate soil sampling locations, and cyan lines show provincial boundaries. The inset in the upper left corner shows the location of the study area within China. Subplots (**a**–**d**) display high-resolution remote sensing images of four representative sampling regions: (**a**) Daan, (**b**) Qianguo, (**c**) Yushu, and (**d**) Gongzhuling. Green dots mark the sampling points in each region. Scale bar is in miles.

This study compiled soil samples collected across Northeast China from May 2017 to May 2024, encompassing three field sampling campaigns. These included 122 samples collected across the northeast in 2017, 499 samples acquired in Si He Town in 2021, and 80 samples collected in Long Zhao Town, Wu Lan Tu Ga Town, and Hei Lin Zi Town in 2024, resulting in a total of 700 soil samples. The spatial distribution of the sampling locations is shown in Figure 1.

**Figure 2:**
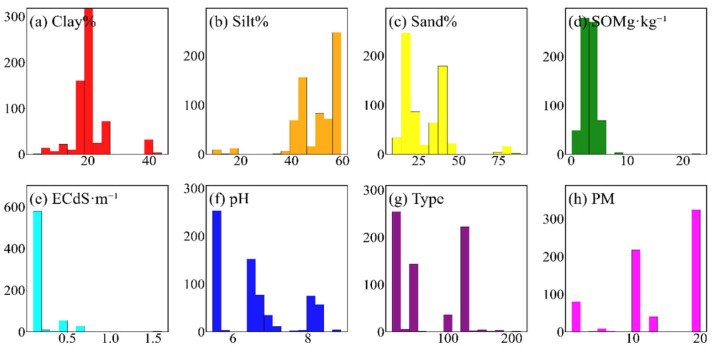
Statistical summary of soil sample properties.

To enhance the representativeness of the ground samples, sampling areas were selected by jointly considering soil types, PM, and other environmental conditions to ensure that the sites captured the spatial variability of typical cropland soils in Northeast China. The sampling points are distributed between latitudes 40.94° N and 46.87° N and longitudes 121.83° E and 130.67° E, covering typical cropland soil types across Liaoning, Jilin, and Heilongjiang provinces, thereby capturing the spatial heterogeneity of typical cropland soils in Northeast China. Within each cropland plot, a 30 m × 30 m square sampling area was established, and surface soil samples from the 0–5 cm layer were collected using the five-point sampling method. The five subsamples were mixed in the laboratory, cleaned of impurities, oven-dried at 85 °C, and sieved through 2 mm mesh. Each mixed soil sample was then divided into two subsamples for laboratory physicochemical property analysis and laboratory spectral measurements, respectively. pH was determined potentiometrically following ISO 10390:2021 [39]. Soil organic matter (SOM) was measured using the external heating potassium dichromate oxidation method [40]. Particle-size fractions were analyzed using the hydrometer method based on ASTM D7928 [41,42]. ECe was determined using the standard saturated soil paste extract method [43]. Figure 2 illustrates the distribution ranges and sample counts of the physicochemical properties. The average clay, silt, and sand contents were 20.93%, 50.40%, and 28.67%, respectively, with ranges of 2.17–43.98%, 9.01–59.46%, and 8.30–88.82%. SOM, EC, and pH exhibited mean values of 3.40 g·kg^−1^, 0.15 dS·m^−1^, and 6.52, with corresponding ranges of 0.12–23.08 g·kg^−1^, 0.01–1.60 dS·m^−1^, and 5.46–8.89. The dominant soil types were albic soil, meadow soil, and chernozem, primarily developed from PM including clay-rich loess derived from coral reef deposits, alluvial–proluvial sediments, and weathered light-colored coarse crystalline rocks.

(2) Indoor soil spectral reflectance

**Figure 3:**
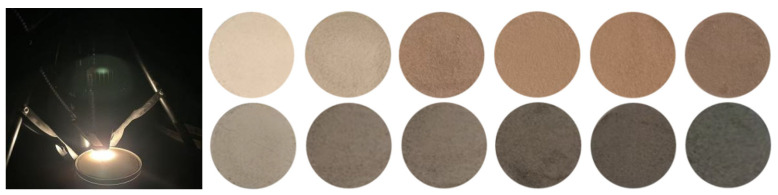
Representative soil samples and measurement methods.

Spectral reflectance measurements of the soil samples were conducted in a dark room using an ASD FieldSpec-4 spectroradiometer (Analytical Spectral Devices, Inc., Boulder, CO, USA) covering the wavelength range of 350–2500 nm. Each soil sample was placed in a black Petri dish with a diameter of 60 mm and a depth of 10 mm, ensuring a uniformly smoothed surface. Figure 3 presents 12 soil samples with distinct color variations, further demonstrating the heterogeneity and representativeness of the dataset used in this study. During spectral acquisition, the fiber-optic probe was mounted vertically at a height of 3 cm above the sample surface to maintain consistent measurement geometry. To minimize measurement errors, the instrument was preheated for 30 min prior to data collection, and white reference calibration was performed every 30 min to suppress signal drift and reduce noise. Each soil sample was measured 10 times at 1-s intervals, and the averaged spectrum was used as the final reflectance for that sample [44]. In total, spectral reflectance measurements were obtained for all 700 soil samples.

#### 2.2.2. ISRIC Data

The International Soil Reference and Information Centre (ISRIC) serves as a global repository of soil spectral and physicochemical property data, integrating soil information from diverse geographic regions worldwide [45]. This database has been extensively utilized in soil spectral modeling and remote sensing inversion studies. Reflectance spectra within the 400–2500 nm range were measured by ISRIC at the ICRAF laboratory using an ASD FieldSpec FR spectroradiometer.

#### 2.2.3. Soil Properties of Cropland in Northeast China

To spatially characterize dry soil reflectance across croplands in Northeast China, this study aims to predict dry soil spectral reflectance using environmental and soil-related covariates. Specifically, ten representative features were selected and processed as input variables.

These features include clay content, silt content, sand content, electrical conductivity (EC), pH, soil type, and parent material (PM), which were derived from the Harmonized World Soil Database version 2.0 (HWSDv2.0) with a spatial resolution of 1 km [46]. In addition, soil organic matter (SOM, g·kg^−1^) was obtained from the China Soil Organic Matter Dataset at the same spatial resolution [47].

Based on these variables, spatial distribution maps of soil properties and environmental covariates were first generated across the Northeast China region. These maps were then integrated and used as model inputs to predict dry soil spectral reflectance at the regional scale. Finally, a continuous spatial distribution map of dry soil reflectance across Northeast China croplands was produced.

All datasets were first reprojected into a unified UTM coordinate reference system and resampled to a spatial resolution of 1 km × 1 km to ensure spatial consistency. Based on the standardized raster data, the geographic coordinates of each pixel center were calculated within the projected coordinate system to generate corresponding longitude (Lon) and latitude (Lat) raster layers. Finally, all ten predictor variables were spatially co-registered and stacked into a single multiband raster dataset for subsequent model input. Utilizing the harmonized spatial resolution and the aligned multiband raster dataset, machine learning algorithms were employed to perform pixel-wise modeling of dry soil reflectance. This approach enabled continuous spatial prediction of dry soil reflectance across the Northeast China cropland region, thereby facilitating accurate simulation of regional-scale soil spectral characteristics.

#### 2.2.4. Sentinel-2 Surface Reflectance

The S2_HARMONIZED reflectance was utilized to generate the maximum reflectance of cropland bare soil, serving as a reference for comparison with the dry soil spectral reflectance simulated by the EEDSR model. The S2_HARMONIZED dataset has undergone comprehensive radiometric, atmospheric, and geometric corrections and is thus directly applicable for compositing. Notably, Band 10 (1375 nm) is exclusively employed for cirrus cloud detection and is excluded from subsequent analysis [48].

Images acquired during the spring tillage period (1 April to 20 May) from 2015 to 2025 were selected. The spring tillage period usually corresponds to the stage when crops have not yet emerged, or vegetation coverage is very low. During this time, the ground surface is mainly bare soil and relatively flat, which is favorable for obtaining comparable bare soil reflectance information at the regional scale. Images with cloud cover exceeding 10% were discarded. Additionally, the Scene Classification Layer (SCL) was used to mask opaque clouds and cirrus clouds, ensuring that extracted pixels were free from cloud contamination. During the temporal compositing of reflectance, the 90th percentile method [49] (P90, defined as the 90th percentile of the reflectance distribution within the time series, denoted as *r_P_*_90_) was applied to mitigate the effects of clouds, haze, crop residues, and sensor uncertainties, thereby producing a robust maximum reflectance composite.

(1) $$r_{P90}(x,b)=Percentile({\left\{r_{t}\left(x,b\right)\right\}}_{t=1}^{T},90)$$

$r_{t}(x,b)$ is the reflectance of pixel x at band b and time *t*, and *T* represents the total number of valid observations for that pixel within the study period. To eliminate the influence of non-cropland and vegetation cover, masking was applied based on the cropland extent derived from the ESA World Cover 2020 dataset [50] combined with a normalized difference vegetation index (NDVI) threshold (NDVI < 0.2) [51]. The composited reflectance was uniformly resampled to a spatial resolution of 1000 m. During resampling, only masked valid pixels were retained; Google Earth Engine automatically excluded NoData during statistical processing, so no interpolation or gap-filling was performed for pixels obscured by clouds or non-cropland soil masks.

#### 2.2.5. Precipitation Data and Surface Soil Moisture Data

The precipitation data were obtained from CHIRPS (Climate Hazards Group infrared precipitation with station data), a daily precipitation product released by the Climate Hazards Group at the University of California, Santa Barbara (UCSB CHG). This dataset integrates satellite remote sensing and ground-based meteorological station observations, providing global coverage with a temporal range spanning from 1981 to 2025. Featuring a spatial resolution of approximately 5 km and a daily temporal resolution, CHIRPS is well suited for regional-scale precipitation analysis [52].

Surface soil moisture data were obtained from the ERA5-Land reanalysis dataset produced by the European Centre for Medium-Range Weather Forecasts (ECMWF). The daily surface soil moisture product for the 0–7 cm soil layer was used, corresponding to the variable volumetric_soil_water_layer_1 (unit: m^3^ m^−3^). ERA5-Land provides spatially and temporally consistent land surface variables by assimilating multi-source meteorological observations within a physically based land surface modeling framework, making it suitable for regional-scale analyses of land surface moisture conditions.ERA5-Land offers global coverage at a spatial resolution of approximately 9 km and a daily temporal resolution, with a continuous temporal record extending from 1981 onward [53].

### 2.3. Method for Modeling Dry Soil Reflectance

**Figure 4:**
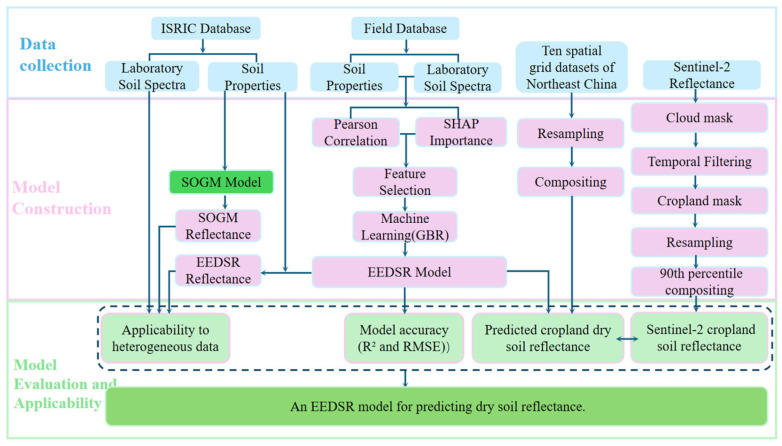
Workflow for the construction and validation of the dry soil reflectance model (EEDSR).

The construction of the dry soil reflectance model in this study primarily involves three parts (Figure 4): data collection, model construction, and model evaluation and applicability. First, soil samples were collected and processed to obtain both soil physical and chemical properties and laboratory-measured spectral reflectance, forming the foundational dataset for this research. Next, correlation and feature importance analyses were conducted between soil properties and soil reflectance to a gradient boosting regression (GBR)-based dry soil reflectance prediction model, termed the environmental and edaphic-factor-driven smooth dry soil reflectance model (EEDSR). Finally, the model’s generalization capability and practical applicability were validated using the ISRIC database. Furthermore, the spatial distribution of the modeled dry soil reflectance for Northeast China (*r_EEDSR_*) was compared with the Sentinel-2 maximum reflectance composite (*r_S2_P90_*) to further demonstrate the model’s suitability and robustness.

#### 2.3.1. Model Indicators Based on Correlation and Importance

Soil physicochemical properties influence surface characteristics that modulate electromagnetic wave scattering and absorption processes at the microscopic scale. These interactions manifest macroscopically as distinct optical behaviors, producing characteristic spectral reflectance curves spanning the VIS to SWIR regions [54]. Based on previous research findings [55], this study selected ten predictive features for the dry soil reflectance model: soil texture components (clay, silt, and sand), SOM, pH, EC, type, PM, and (Lon, Lat). To quantitatively assess the relationships between soil features and spectral reflectance, Pearson correlation coefficients (R) were computed between each soil property and reflectance across the 350–2500 nm wavelength range. The R at each wavelength was calculated using the formula

(2) $$R_{X_{j},r(\lambda )}=\frac{\sum_{i=1}^{n}\left(X_{i,j}-\bar{X_{j}})(r_{i}\left(\lambda \right)-\bar{r(\lambda )}\right)}{\sqrt{\sum_{i=1}^{n}{\left(X_{i,j}-\bar{X_{j}}\right)}^{2}}\cdot \sqrt{\sum_{i=1}^{n}{\left(r_{i,}(\lambda )-\bar{r(\lambda )}\right)}^{2}}}$$

where $R_{X_{j},r(\lambda )}$ is the Pearson correlation coefficient between the *j*th soil attribute and the reflectance at wavelength $\lambda ;\;X_{i,\;j}$ represents the value of the *j*th soil attribute for the *i*th sample (j = 1, 2, …, 10); $r_{i}\left(\lambda \right)$ is the reflectance of the *i*th sample at wavelength λ. $\bar{X}$*_j_* and $\bar{r(\lambda )}$ are the mean values of the *j*th soil attribute and the reflectance at wavelength λ, respectively;and the total number of samples is denoted by *n*.

In addition, the Shapley Additive exPlanations (SHAP) method was employed to analyze the importance and average contribution of each feature to the dry soil reflectance, thereby identifying key driving factors and elucidating their underlying mechanisms [56].

#### 2.3.2. Environmental and Edaphic-Factor-Driven Smooth Dry Soil Reflectance Model (EEDSR)

Informed by the results of the Pearson correlation analysis and SHAP importance, the ten features described in Section 2.3.1 were selected as input variables to predict dry soil reflectance at different wavelengths. A gradient boosting regression (GBR) framework was applied to develop an environmental and edaphic-factor-driven smooth dry soil reflectance model (EEDSR). Independent models were established at 10 nm intervals spanning 350–2500 nm, resulting in 215 wavelength-specific sub-models. The dataset was randomly split into training (70%) and test (30%) subsets. To mitigate potential bias arising from uneven sample distribution, a stratified sampling strategy based on electrical conductivity (EC) was applied during model training to ensure balanced representation and thus minimize its impact on model development. Hyperparameters were optimized on the training set via grid search combined with 10-fold cross-validation. For the full-spectrum 215 sub-models, hyperparameters were independently tuned, and Table 1 presents the hyperparameter ranges derived from Sentinel-2 central wavelengths. To balance computational efficiency and modeling consistency, the hyperparameter search space was constrained based on the Sentinel-2 optimization results. These bands cover the visible, near-infrared, and shortwave infrared regions and effectively represent the key spectral domains governing soil reflectance variability, making the parameter ranges transferable for full-spectrum optimization The search space included the number of boosting trees (n estimators = 900, 1100, 1300), learning rate (0.01, 0.05, 0.1), maximum tree depth (max depth = 5, 6, 7), minimum samples to split an internal node (min samples split = 2, 5, 10), and minimum samples per leaf (min samples leaf = 1, 2, 4). Each wavelength-specific sub-model was independently optimized within this constrained search space. The hyperparameter set yielding the best average cross-validation performance was selected for final model construction. Model performance was evaluated on the independent test set using the coefficient of determination (R^2^) and root mean square error (RMSE), quantifying the accuracy and robustness of dry soil reflectance predictions across the spectrum.

### 2.4. Accuracy Evaluation of EEDSR

To validate the predictive reliability and applicability of the proposed EEDSR model on heterogeneous data, the SOGM was employed as a benchmark for comparison. SOGM is a physics-informed, data-driven generative framework that synthesizes dry soil reflectance spectra from the visible (VIS) to the shortwave infrared (VIS–SWIR) region based on soil physicochemical properties. It integrates spectral embedding encoding with a diffusion-based reconstruction process to generate soil reflectance spectra [28]. Trained on a comprehensive global database comprising approximately 180,000 samples from 17 soil spectral datasets, SOGM has demonstrated strong capability in spectral generation and reconstruction, effectively capturing complex nonlinear relationships between soil attributes and spectral responses. To ensure compatibility with model input requirements and to enable a fair and consistent comparison under heterogeneous conditions, a total of 70 samples were selected from the ISRIC global soil database These samples were collected from six countries, China (*n* = 20), the United States (*n* = 15), Hungary (*n* = 10), Zambia (*n* = 8), Oman (*n* = 7), and Romania (*n* = 10), representing diverse soil types and environmental settings distinct from the regional training domain of EEDSR. All selected samples simultaneously contain complete soil physicochemical properties (clay, silt, sand, SOM, pH, and EC) and measured spectral reflectance data spanning the 400–2500 nm wavelength range.

Both EEDSR and SOGM were evaluated under identical input constraints, using the same set of soil properties, the same spectral range (400–2500 nm), and an identical sample partitioning strategy. Model-predicted dry soil reflectance spectra were compared against the corresponding ISRIC reference spectra, thereby establishing an independent and heterogeneous validation framework for assessing model generalizability and applicability across different geographic and environmental conditions.

## 3. Results

### 3.1. Pearson Correlation and SHAP-Based Importance of Model Indicators

**Figure 5:**
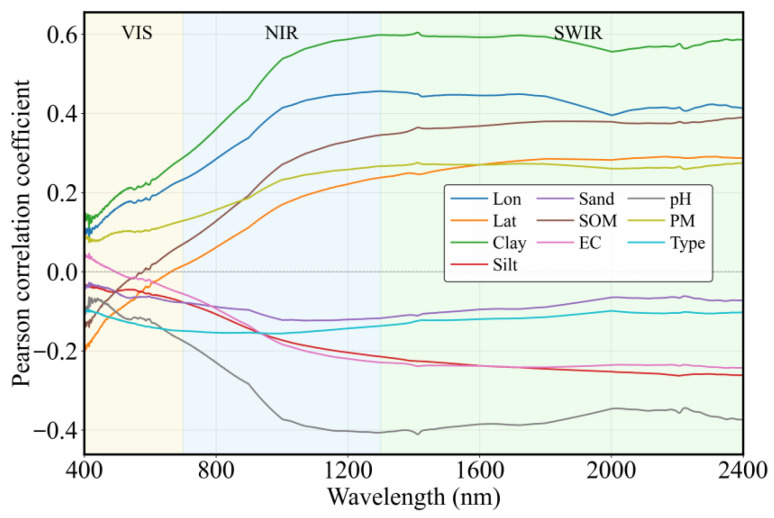
Correlation analysis between ten soil properties and spectral reflectance.

The ten features exhibit varying Pearson correlation relationships with dry soil reflectance across the 350–2500 nm spectral range (Figure 5). Clay, longitude (Lon), and parent material (PM) exhibit positive correlations with reflectance throughout the spectral range, with correlation strength increasing as wavelength increases. Conversely, silt, sand, pH, and soil type generally show negative correlations. Among these, the absolute correlations of silt and pH increase with wavelength, while sand and soil type show minimal variation across wavelengths. Soil organic matter (SOM) and latitude (Lat) are negatively correlated with reflectance in the visible (VIS) region but present weak positive correlations in the NIR–SWIR. EC shifts from a positive correlation in the VIS region to a weak negative correlation in the NIR–SWIR regions. Overall, these results indicate that the correlations between soil features and spectral reflectance are wavelength-dependent, highlighting the significant influence of wavelength on soil spectral response patterns.

**Figure 6:**
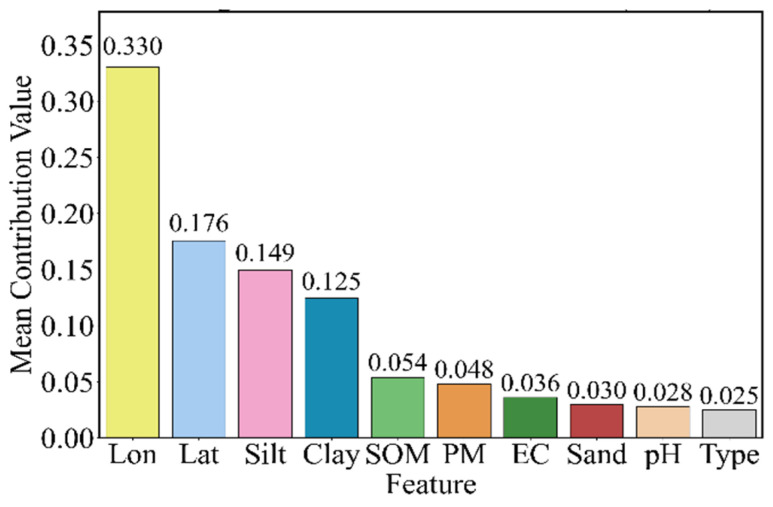
Mean contribution of ten selected features to dry soil reflectance based on SHAP analysis.

Based on the Pearson correlation analysis, the SHAP (Shapley Additive exPlanations) method was further employed to assess the overall importance and contribution of these features to dry soil reflectance prediction (Figure 6). The results indicate that all features contribute to the prediction of dry soil reflectance, with their importance ranked from highest to lowest as follows: Lon, Lat, silt, clay, SOM, PM, EC, sand, pH, and type. Spatial factors (Lon and Lat) exhibit the most significant contributions, followed by soil texture components, and finally soil chemical properties and soil type. Overall, the selected ten features demonstrate strong rationality, as validated by both correlation and importance analyses. These findings reveal the spectral band dependency and multifactorial drivers of dry soil reflectance, providing a solid theoretical and empirical foundation for the construction of dry soil reflectance models.

### 3.2. Performance of Dry Soil Reflectance Using the EEDSR Model

**Figure 7:**
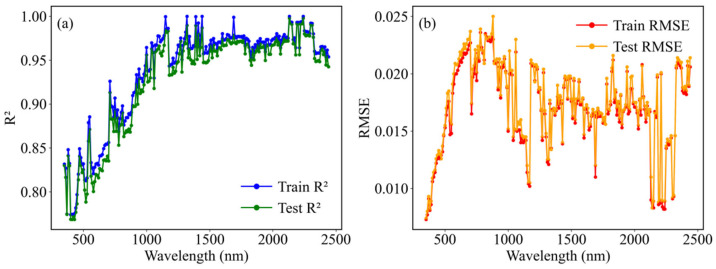
The R^2^ (**a**) and RMSE (**b**) of dry soil reflectance at 10 nm intervals (350–2500 nm) using the EEDSR model with ten features.

The evaluation of the EEDSR model’s performance on both training and testing datasets reveals that the R^2^ initially starts low then increases and stabilizes as the wavelength progresses across the VIS–SWIR spectral regions (Figure 7a). The RMSE remains relatively low at shorter wavelengths, rises notably between approximately 600 and 1000 nm, and subsequently decreases, maintaining a low level at longer wavelengths (Figure 7b). Overall, the EEDSR model exhibits high fitting accuracy and stability, achieving an average R^2^ of 0.93 and an average RMSE of 0.018 over the 400–2500 nm wavelength range for both datasets.

**Figure 8:**
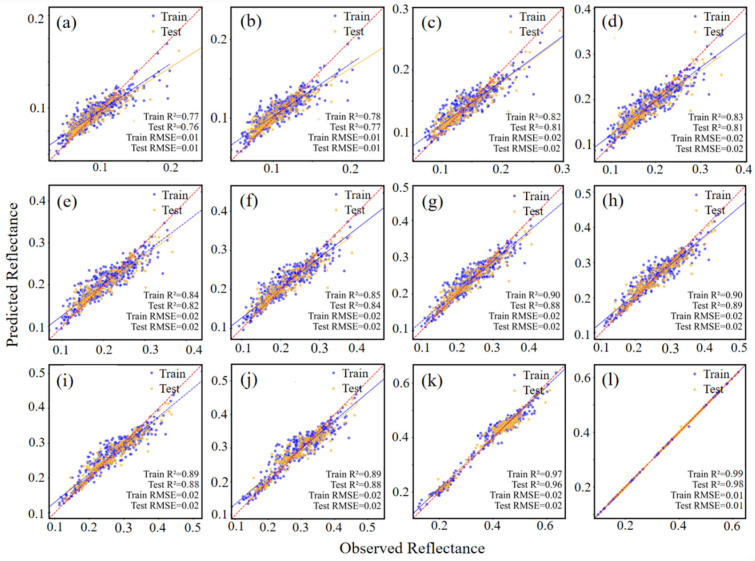
Scatter plot of dry soil spectral reflectance based on the EEDSR model for Sentinel-2 bands: (**a**) 443nm, (**b**) 490 nm, (**c**) 560 nm, (**d**) 665 nm, (**e**) 705 nm, (**f**) 740 nm, (**g**) 783 nm, (**h**) 842 nm, (**i**) 865 nm, (**j**) 945 nm, (**k**) 1610 nm, (**l**) 2190 nm.

The predictive performance of the EEDSR model for dry soil reflectance was assessed across 12 Sentinel-2 spectral bands using both training and testing datasets. Within the visible (VIS) bands (443, 490, 560, and 665 nm; Figure 8a–d), the coefficient of determination (R^2^) ranged from 0.76 to 0.83, with root mean square error (RMSE) values between 0.01 and 0.02. Enhanced performance was observed in the red-edge bands (705, 740, and 783 nm; Figure 8e–g), where R^2^ generally exceeded 0.84 and RMSE remained near 0.02. In the near-infrared (NIR) bands (842, 865, and 945 nm; Figure 8h–j), R^2^ further increased, consistently surpassing 0.88, accompanied by RMSE values within the 0.01 to 0.02 range. The shortwave infrared (SWIR) bands (1610 and 2190 nm; Figure 8k,l) exhibited the highest fitting accuracy, with R^2^ exceeding 0.96 and RMSE stabilized at low levels. Together, these findings demonstrate the robustness and reliability of the EEDSR model across the multispectral Sentinel-2 bands, highlighting the potential of machine learning approaches for dry soil reflectance modeling.

### 3.3. Extrapolation Ability of EEDSR Model: Comparison with SOGM

**Figure 9:**
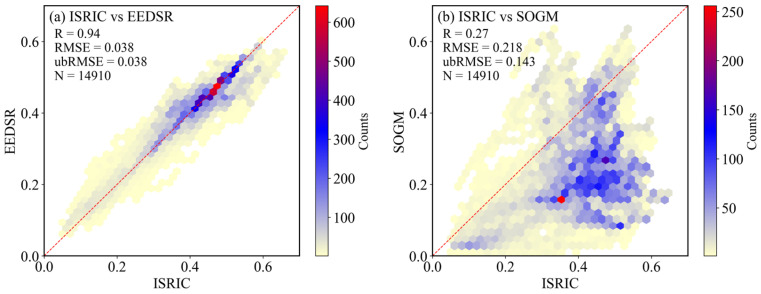
Comparison between the EEDSR model and the SOGM based on the ISRIC database.

Under identical input conditions, the dry soil reflectance simulations of EEDSR and the SOGM were compared using the ISRIC database (Figure 9). Although the complete training conditions of the SOGM were not fully replicated in this study, a direct performance comparison under consistent input parameters was achievable. The results demonstrate that EEDSR exhibits a significantly higher correlation coefficient with measured reflectance (R = 0.94,RMSE = 0.038, ubRMSE = 0.038) compared to the SOGM (R = 0.27, RMSE = 0.218, ubRMSE = 0.143). The EEDSR shows superior fidelity in reproducing observed soil reflectance, whereas the SOGM presents certain biases, likely attributable to limited input attributes. Overall, these findings indicate that the proposed EEDSR possesses strong generalization ability and promising applicability across heterogeneous datasets.

## 4. Discussion

### 4.1. Regulatory Effect of Parent Material and Geolocation on Predicting Dry Soil Reflectance

The results of this study indicate that dry soil reflectance exhibits significant spatial variability at the regional scale, highlighting a clear spatial dependence. Longitude and latitude, as proxies of spatial position, can effectively capture environmental gradients and have been widely used in digital soil mapping to represent variations in climate and topography [57]. These environmental gradients influence soil physical and chemical properties, which in turn contribute to the spatial variability of dry soil reflectance [52]. Specifically, changes in latitude lead to significant differences in solar radiation intensity and day length, resulting in distinct latitudinal gradients in temperature and precipitation [58]. Geographic variables such as latitude represent environmental gradients rather than direct drivers of spectral reflectance. These gradients are associated with variations in climate and topography, which influence soil formation processes and soil physicochemical properties. It is these soil properties that ultimately determine the spectral response characteristics of dry soils. These climatic conditions affect soil organic matter content, moisture status, and mineral composition [59]. Changes in longitude reflect differences in continental and oceanic distributions, giving rise to different climate types; regions closer to oceans typically have higher humidity and precipitation, whereas inland areas tend to be drier. Additionally, longitudinal differences can influence prevailing wind directions and airflow patterns, thereby regulating soil moisture and salt accumulation [60]. These influences are consistent with classic soil-forming factor paradigms where climate and topography play dominant roles in shaping soil properties across landscapes [61].

**Figure 10:**
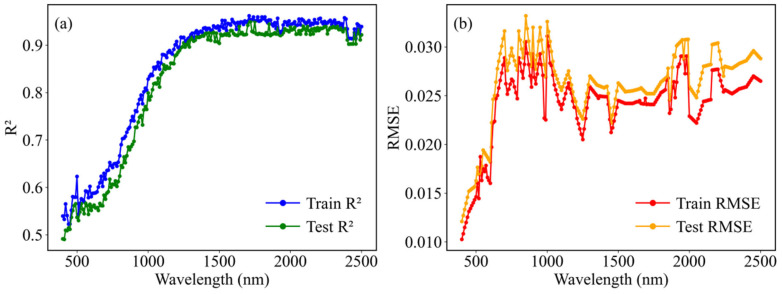
The R^2^ (**a**) and RMSE (**b**) of dry soil reflectance at 10 nm intervals (350–2500 nm) using the EEDSR model with seven features without Lon, Lat, and PM.

Climate and topography, as key environmental factors closely related to longitude and latitude, determine the spatial distribution patterns of PM. Parent material determines the initial soil texture, while subsequent pedogenetic processes may, over time, alter particle-size distribution and soil structure. Soil parent material is a key factor influencing the initial conditions of soil formation; however, soil texture can also be modified by pedogenetic processes, such as the translocation of clay and silt within the soil profile [62]. These processes jointly affect soil structure and light-scattering properties, thereby influencing soil structure and spectral reflectance characteristics [63]. Furthermore, parent material influences soil weathering stages, pH values, and organic matter accumulation capacity, all of which play important roles in the spectral response mechanisms of dry soils. The model performance after removing longitude, latitude, and parent material as features shows an average R^2^ of 0.82 (Figure 10). In contrast, the full-feature model achieves an R^2^ of 0.93 (Figure 7), indicating a significant decline in prediction accuracy after excluding these key spatial variables. In summary, the physical, chemical, and biological properties of soils exhibit regular zonal or regional patterns in geographic space, a phenomenon known as “zonal distribution” or “soil zonality” [64]. This pattern is primarily driven by natural factors such as climate, topography, and parent material.

The model performance after removing longitude, latitude, and parent material as features shows an average R^2^ of 0.82 (Figure 10). In contrast, the full-feature model achieves an R^2^ of 0.93 (Figure 7). The results indicate that including parent material and geographic features significantly improves model performance, and the feature importance ranking also highlights the significance of longitude and latitude.

### 4.2. Applicability of EEDSR Model in Northeast China Cropland: Comparison with Sentinel-2 Reflectance

**Figure 11:**
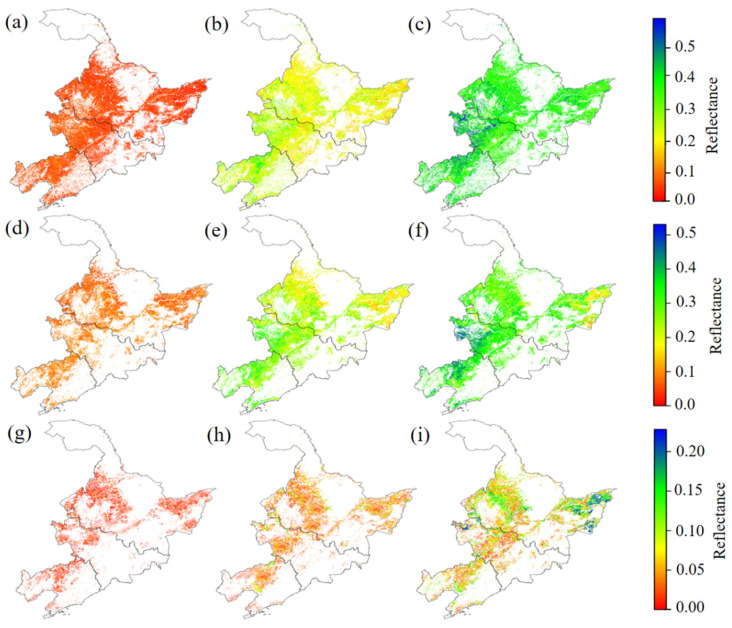
The spatial distribution of simulated dry soil reflectance from EEDSR model, Sentinel-2 maximum soil reflectance, and their bias for the crop regions of Northeast China. (**a**–**c**): Predicted dry soil reflectance (r*_EEDSR_*), (**d**–**f**): Sentinel-2 P90 cropland bare soil reflectance (r*_S2_P90_*), (**g**–**i**): Spatial distribution of the bias between r*_EEDSR_* and r*_S2_P90_*.

**Figure A1:**
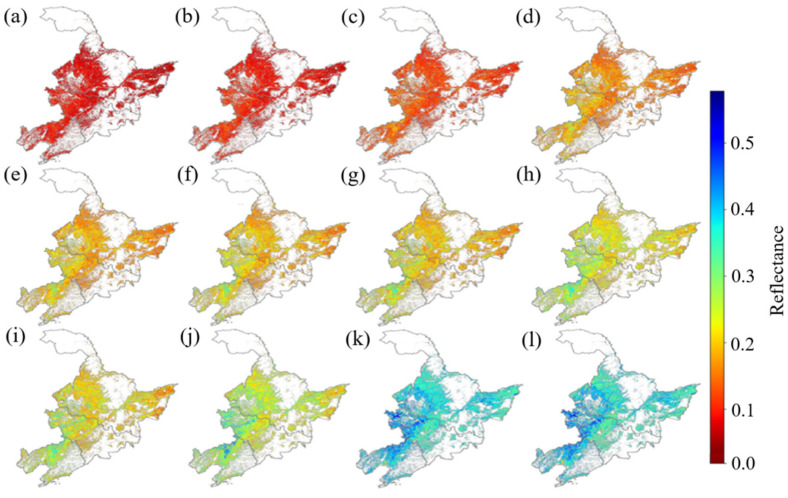
The dry soil reflectance in cropland bare soil regions was predicted by the EEDSR model. (**a**–**l**) The band central wavelengths of Sentinel-2 bands: (**a**) 443 nm, (**b**) 490 nm, (**c**) 560 nm, (**d**) 665 nm, (**e**) 705 nm, (**f**) 740 nm, (**g**) 783 nm, (**h**) 842 nm, (**i**) 865 nm, (**j**) 945 nm, (**k**) 1610 nm, (**l**) 2190 nm.

Based on the approach detailed in Section 2.2.3, the spatial distribution of predicted dry soil reflectance (*r_EEDSR_*) at Sentinel-2 central wavelengths of 443 nm, 842 nm, and 1610 nm was mapped across the cropland areas in Northeast China (Figure 11a–c). The dry soil reflectance exhibits a wavelength-dependent trend, initially low in the VIS range (400–700 nm), followed by a marked increase entering the NIR region (700–1300 nm). In the SWIR region (1300–2500 nm), the reflectance curve displays pronounced fluctuations due to characteristic absorptions by hydroxyl groups, clay minerals, and carbonates, with notable absorption features near 1400 nm, 1900 nm, and 2200 nm, resulting in an overall decreasing trend (Figure A1), with higher reflectance in the western regions compared to the eastern regions.

This spatial variability is primarily driven by the distribution of soil physicochemical properties. Soils in northwestern Northeast China have a high sand content, low water retention capacity, and high salinity, resulting in increased reflectance in the NIR and SWIR bands. In contrast, soils in the southeastern region have high SOM content and dark color, which enhance absorption in the VIS and NIR bands, thereby reducing reflectance.

**Figure A2:**
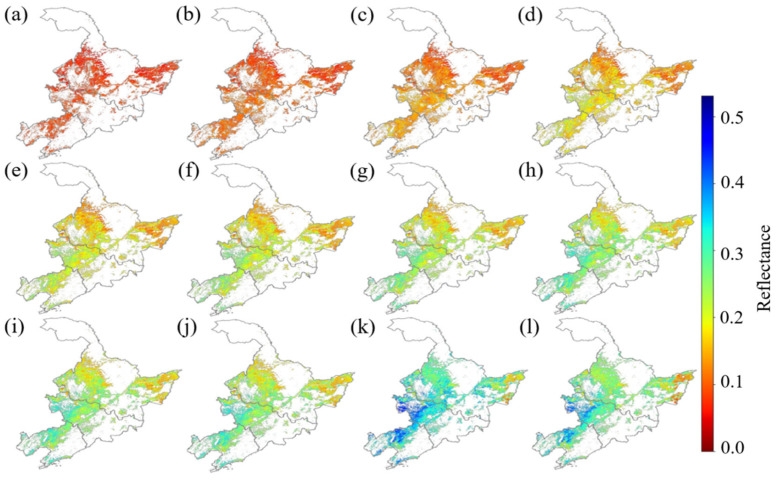
Spatial distribution of the Sentinel-2 P90 reflectance. (**a**–**l**) The band central wavelengths of Sentinel-2 bands: (**a**) 443 nm, (**b**) 490 nm, (**c**) 560 nm, (**d**) 665 nm, (**e**) 705 nm, (**f**) 740 nm, (**g**) 783 nm, (**h**) 842 nm, (**i**) 865 nm, (**j**) 945 nm, (**k**) 1610 nm, (**l**) 2190 nm.

**Figure A3:**
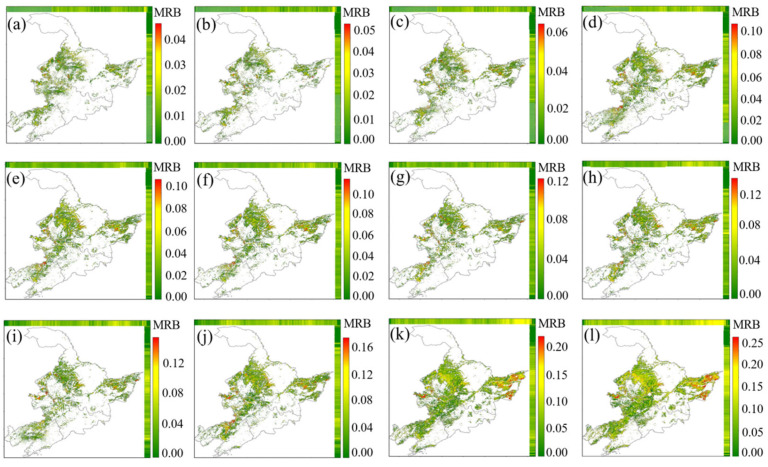
Spatial distribution of the difference between EEDSR-predicted dry soil reflectance and Sentinel-2 P90 reflectance, with the strip gradient representing the average bias. MRB: Mean Reflectance Bias. (**a**–**l**) The band central wavelengths of Sentinel-2 bands: (**a**) 443 nm, (**b**) 490 nm, (**c**) 560 nm, (**d**) 665 nm, (**e**) 705 nm, (**f**) 740 nm, (**g**) 783 nm, (**h**) 842 nm, (**i**) 865 nm, (**j**) 945 nm, (**k**) 1610 nm, (**l**) 2190 nm.

Sentinel-2 P90 cropland bare soil reflectance (*r_S_*_2*_P*90_) at Sentinel-2 central wavelengths of 443 nm, 842 nm, and 1610 nm is shown in Figure 11d–f. The *r_S_*_2*_P*90_ is generally lower than *r_EEDSR_* but exhibits consistent spectral patterns and spatial distributions, indicating a strong correspondence in spectral response characteristics and geographic differentiation between the two datasets (Figure A2). The spatial distribution of the bias between *r_EEDSR_* and *r_S_*_2*_P*90_ at 443 nm, 842 nm, and 1610 nm is shown in Figure 11g–i. Overall, the bias increases progressively from west to east, with the average bias in the eastern cropland soil areas being higher than that in the western regions (Figure A3).

**Figure 12:**
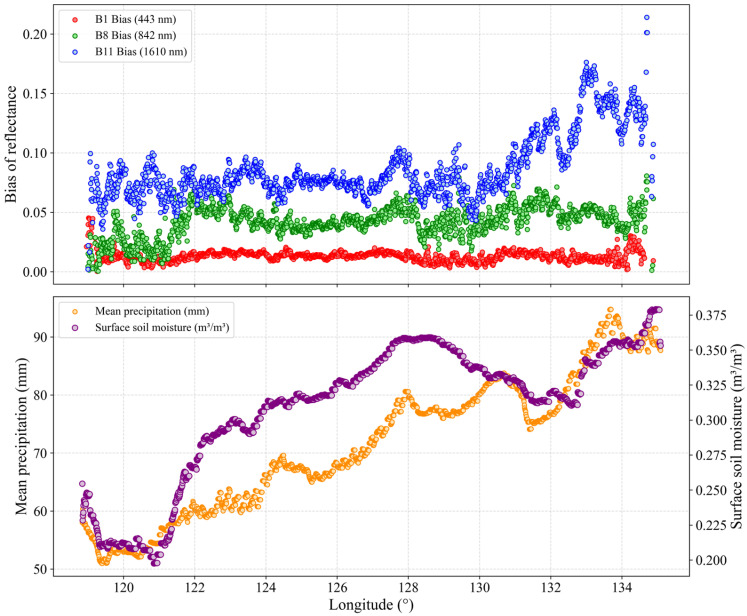
The trends of dry soil reflectance predicted by the EEDSR model, Sentinel-2 P90 reflectance, and the average daily precipitation from 10 April to 20 May 2015–2025, with longitude, for the Sentinel-2 central bands at 443 nm, 842 nm, and 1610 nm.

The longitudinal variation in the average dry soil reflectance bias at the 443 nm, 842 nm, and 1610 nm bands, along with the mean precipitation calculated from cumulative rainfall data between 1April and 20 May during 2015–2025, is presented (Figure 12); the detailed methodology is described in Section 2.2.5. This spatial differentiation confirms the influence of surface soil moisture and precipitation-driven soil moisture patterns on reflectance [65]. In the eastern region, higher precipitation leads to elevated soil moisture, resulting in a wetter and darker soil surface, which causes the *r_S_*_2*_P*90_ to be underestimated relative to the *r_EEDSR_*, thereby increasing the discrepancy between the two. Conversely, soils in the western region tend to be drier, with localized salinization enhancing surface reflectance and causing *r_S_*_2*_P*90_ to align more closely with *r_EEDSR_*.

## 5. Conclusions

This study emphasizes the modeling of dry soil surface spectral reflectance by developing an intelligent learning model (EEDSR) targeted at the Northeast China black soil region. The model comprehensively incorporates multiple soil properties (sand, clay, silt, SOM, pH, EC, type, and PM) along with geographic coordinates to predict dry soil reflectance across the 400–2500 nm spectral range. The key findings regarding model accuracy and evaluation are summarized as follows:

1. The EEDSR model achieved an average R^2^ of 0.93 and RMSE of 0.018 across the 400–2500 nm spectral range.
2. This study incorporates parent material (PM) and geographic coordinates (longitude and latitude) as auxiliary predictive variables for soil reflectance modeling. The inclusion of PM improved the average R^2^ from 0.82 to 0.93 (13.4%).
3. Independent validation using the ISRIC global soil database further confirmed the superior accuracy and generalizability of the EEDSR model (R = 0.94) compared to the existing Soil Optical Generation Model (SOGM, R = 0.27).
4. The spatial distribution patterns of dry soil reflectance predicted by EEDSR closely correspond with the Sentinel-2 maximum reflectance of cropland bare soil, with bias gradually increasing from west to east, consistent with the spatial variability of surface precipitation. This correspondence indirectly validates the rationality of the proposed EEDSR model.

By leveraging machine learning techniques, the EEDSR model effectively establishes the relationship between soil properties and spectral reflectance, providing a solid technical foundation for soil spectral modeling.

Future work should consider incorporating elevation and terrain-related factors as an important extension to better capture soil spatial heterogeneity. In addition, integrating variables such as soil moisture, surface roughness, and observation geometry would further enhance the consistency with satellite-based observations and improve the accuracy of soil reflectance simulation.

## Figures and Tables

**Table 1:** Optimal parameters (based on Sentinel-2 central wavelengths).

Parameter	n Estimators	Learning Rate	Max Depth	Min Samples Split	Min Samples Leaf
443 nm	900	0.01	5	10	4
490 nm	900	0.01	5	10	4
560 nm	900	0.01	5	10	1
665 nm	900	0.01	5	10	4
705 nm	900	0.01	5	2	4
740 nm	900	0.01	5	2	4
783 nm	900	0.01	6	10	4
842 nm	1100	0.01	5	5	2
865 nm	900	0.01	5	5	5
945 nm	900	0.01	5	5	2
1375 nm	900	0.01	6	2	2
1610 nm	1100	0.01	5	10	2
2190 nm	900	0.05	7	2	1

## Data Availability

The data used to support the findings of this study are available from the corresponding author upon request.
